# Traces of epidemics in the fringes of pain: memory of Spanish flu from a local history perspective, Botucatu (São Paulo state), 1918

**DOI:** 10.1590/S0104-59702023000100061

**Published:** 2023-11-10

**Authors:** Anna Cristina Rodopiano de Carvalho Ribeiro, Maria Cristina da Costa Marques

**Affiliations:** i Doutoranda, Programa de Pós-graduação em Saúde Pública/Faculdade de Saúde Pública/Universidade de São Paulo. São Paulo – SP – Brasil criscarvalho2000@usp.br; ii Professora, Programa de Pós-graduação em Saúde Pública/Faculdade de Saúde Pública/Universidade de São Paulo. São Paulo – SP – Brasil mcmarques@usp.br

**Keywords:** Epidemics, Influenza pandemic 1918-1919, Public health/history, Public health profiles/history, Brazil, Epidemias, Influenza pandêmica 1918-1919, Saúde pública/história, Perfis sanitários/história, Brasil

## Abstract

This article takes a local history perspective to scrutinize how the memory of suffering that surrounded the Spanish flu epidemic of 1918 in Botucatu, São Paulo state, has been evoked, challenged, and transmuted over time, producing representations in strategies and practices, and understandings that end up constituting a meaning-making social reality. In this historiographic endeavor, historical vestiges were brought together from a variety of the city’s archives between September and October 2021 in a bid to reveal the historical processes that were accreted and deposited in the social fabric and fibers, and which, under the processes of time, were changed and reworked, bringing forth the ineffable mark of Spanish flu.

## Resumo

O artigo perscruta sob a perspectiva da história local de que forma a memória do sofrimento que cercou a epidemia de gripe espanhola de 1918 em Botucatu (interior paulista) foi reconvocada, tensionada e transmutada ao longo do processo histórico, produzindo representações em estratégias e práticas, e apreensões como constituintes de uma realidade social que produz sentidos. Para essa operação historiográfica, coligiram-se vestígios históricos em diversos arquivos botucatuenses, entre setembro e outubro de 2021, buscando desvelar processos históricos aglutinados e depositados entre as fibras e fímbrias sociais e que, sob afecção da temporalidade, se movimentam, se reelaboram e trazem à tona a inefável marca da gripe espanhola.

Segundo Marques (2018, p.3), o pensamento histórico na interpretação dos eventos e fenômenos em estudos de saúde coletiva articulados aos seus contextos nos permite compreender mecanismos de racionalidade que possibilitaram respostas e políticas de atenção, ressurgência de antigos males e emergência de novos riscos sanitários. No entanto, se faz fundamental atentar à armadilha da "má história", em que o argumento histórico se faz descritivo, enaltecedor e/ou acrítico, levando à produção historiográfica eivada, pautada em continuísmo e "lições do passado" (Berridge, 2010, p.798).

Tal cenário nos aponta o imperativo de desnaturalizar e historicizar vasto campo de acontecimentos que a pandemia de covid-19 dramaticamente nos oferta. Entre eles, a intensa evocação à epidemia de gripe espanhola por diversos atores sociais e o que a apreensão de contextos, representações, tensões e silenciamentos na rememoração desse flagelo podem nos revelar sobre os recônditos da experiência humana (Marques, Ribeiro, Mota, 2021; Silveira, Nascimento, 2018; Alvarez et al., 2009).

Para Jean Delumeau (2009), que se deteve sobre o imaginário humano diante do medo no Ocidente entre os séculos XIII e XVIII, haveria uma tipologia de comportamentos coletivos e representações mentais comuns diante da ameaça da peste com a negação de sua existência. Quando já não se podia esconder a ocorrência epidêmica, não raras as vezes, o diagnóstico de facultativos era tranquilizador, ainda que o quadro apontasse o contrário.

Reis (1991), em análise histórica sobre ritos fúnebres e revolta popular no Brasil do século XIX, se refere ao apagamento dos sinais da morte como recurso médico para escamotear a impossibilidade de evitá-la.

Já Claúdia Rodrigues (1999) debruça-se sobre mudanças advindas do impacto da febre amarela e sua mortandade no Rio de Janeiro de 1850. Segundo a estudiosa, o medo do contágio desdobrou-se no temor aos finados, transformando rituais e propiciando a implementação de concepções médicas sobre cadáveres, sepulturas e enterramentos.

Jacques Le Goff (1991, p.8), por sua vez, nos atenta para a história dramática do sofrimento e da febre conjuntural das epidemias que se desenrola ao longo dos tempos e articula "o horror dos sintomas ao pavor de um sentimento de culpabilidade individual e coletiva".

Charles Rosenberg (1989), ao estudar a materialidade social das epidemias, propõe a dramaturgia epidêmica. Nessa dramaturgia, o desenrolar da doença seria marcado por atos contínuos em crescente tensão, compondo narrativa repetitiva em certos padrões de episódios passados, ofertando-nos quadro teórico-analítico que apontaria propensão à reincidência de percepções ou reações diante do adoecimento coletivo (Rosenberg, Golden, 1992).

Defende-se, todavia, que "a especificidade importa mais do que a repetição" (Chalhoub, 2006, p.515). Ou seja, para além dos traços partilhados, acredita-se que a investigação historiográfica necessita reter o que tais experiências epidêmicas carregam de peculiar e imprevisto, revelando marcas desse viver social dramático.

Nessa medida, considerando que a dor significa, assim como sua recusa ou captura (Farge, 2011, p.9), este artigo assenta-se na dialética das durações do tempo e na perspectiva da história local, propondo-se prescrutar como a memória da gripe espanhola em Botucatu (município do interior paulista) foi reconvocada, tensionada e transmutada ao longo do processo histórico.

A história local, como campo de saber em diálogo político e teórico com a história social, é aqui entendida como investigação histórica de tema-problema restrito a espaço físico-político-social (Marson, 2020, p.46), permitindo mediatizar ocorrências particulares e pressupostos universais que organizam o mundo contemporâneo (Robin, 2016, p.185; Marson, 2020, p.35).

Desse modo, a partir de pesquisa empírica realizada em seis arquivos históricos e um acervo pessoal em Botucatu nos meses de setembro e outubro de 2021, o trabalho pretende desvelar processos históricos aglutinados entre fibras e fímbrias sociais, que sob afecção da temporalidade se movimentam e se reelaboram (Braudel, 1992; Le Goff, 2003; Vovelle, 1998; Chartier, 1989, p.9) trazendo à tona marcas da gripe espanhola.

## A febre conjuntural de 1918

Considerada por um século a maior epidemia da história devido a sua alta letalidade em curto interstício, a gripe espanhola atingiu de 80% a 90% da população do planeta, associando-se rapidamente a graves infecções respiratórias na sequência da contaminação gripal (Frada, 2005, p.18).

Acredita-se que a epidemia tenha se originado em campos de treinamento militar nos EUA, e que sua alcunha se deva ao fato de a Espanha, neutra na Primeira Guerra Mundial, ter veiculado em sua imprensa a existência da enfermidade, levando à associação dos primeiros casos da doença a este país (Bertucci, 2005b, p.155).

Propagando-se pelos continentes com o deslocamento de tropas, a gripe espanhola, na primavera de 1918, invadiu o Brasil, na época envolvido com o saneamento rural e a questão nacional, onde a interiorização da saúde regeneraria corpos e conferiria um povo à nação (Hochman, 1998; Marques, Ribeiro, Mota, 2021, p.235).

Ao aportar inicialmente no Rio de Janeiro – então capital do Brasil –, a bordo do navio *S.S. Demerara*, vindo de Liverpool, em 14 de setembro de 1918, depois de fazer escalas em Lisboa, Recife e Salvador, a gripe espanhola alastrou-se rapidamente pelo território brasileiro, descerrando fragilidades na defesa sanitária e tensionando os saberes médicos como prática oficial de cura (Bertolli Filho, 2003; Bertucci, 2004; Chagas Filho, 1993; Silveira, Nascimento, 2018).

No estado de São Paulo, o Serviço Sanitário paulista confirmou a epidemia oficialmente à população em 15 de outubro de 1918, com auge epidêmico entre 23 de outubro e o transcorrer de novembro de 1918, e declínio a partir do dia 26 deste último mês.

Nesse ínterim, os boletins de Arthur Neiva, diretor geral do Serviço Sanitário paulista, a instalação de telefones para orientações e relatos, e a convocação de municipalidades e de setores da sociedade pelo secretário de Estado de Negócios do Interior foram algumas das medidas tomadas pelo Poder Público. Tais ações, entretanto, não impediram que a crise sanitária e a convulsão social se instalassem na capital paulista com desabastecimento, saques e cadáveres aguardando enterramentos (Meyer, Teixeira, 1920; Bertucci, 2004; Bertolli Filho, 2003).

À desestruturação das autoridades municipais e sanitárias – onde se fez imprescindível a organização popular no socorro à população –, somou-se a sobreposição de tragédias:

em delírio febril, gripados atentavam contra a própria vida ou roubavam a existência de outrem (Bertolli Filho, 2003; Bertucci, 2004).

Em meio a tantas tentativas de burlar a epidemia, a administração intensa de purgantes provocava frequentes desmaios pelas ruas, confundindo-se com os próprios sintomas da gripe. Aos sobreviventes, sobravam ofertas de compostos e acessórios para solucionar ou disfarçar a queda de cabelos que os atacava na convalescença gripal (Ribeiro, 2020).

Em São Paulo, sem considerar as diferenças abissais entre as condições materiais de vida próprias a cada grupo social e que determinavam desiguais oportunidades de viver e morrer durante a epidemia, as autoridades sanitárias do período propugnavam a ilusão democrática da gripe. Nesse discurso, a semelhança biológica da população possibilitava que todos os paulistanos fossem igualmente susceptíveis à infecção gripal ou a ela sobrevivessem ou não (Bertolli Filho, 2003).

No cotejamento do perfil patológico da cidade com as particularidades dos distritos paulistanos e a propagação da doença, contudo, Bertolli Filho (2003) concluiu que os maiores focos de infecção epidêmica se encontravam em bairros periféricos e com aparato urbano e sanitário precário ou inexistente.

Até o último dia de 1918, somaram-se oficialmente 116.777 moradores gripados e 5.331 mortes na cidade de São Paulo, ou seja, 1% da população da capital – que na época somava pouco mais 528 mil habitantes – não inclusos os adoecidos com sequelas e complicações (Bertucci, 2004). Bertolli Filho (2003, p.85), por seu turno, chegou em seus estudos ao número de 5.429 mortes na pauliceia.

Há muito ainda a se desvendar sobre a passagem da gripe espanhola e seus impactos no Brasil, pois, comumente, as investigações restringem-se à passagem da epidemia nas capitais e cidades litorâneas do país. Nesse sentido, relevante destacar os estudos de Bertucci (2005a) e Dall'Ava (2017) que, ao analisarem as estratégias e particularidades dos municípios paulistas de Campinas e Sorocaba, respectivamente, no enfrentamento à gripe espanhola, forneceram fundamental contribuição à história regional de São Paulo.

Desta feita, o estudo sobre os desdobramentos da gripe espanhola em Botucatu apresenta-se instigante ao ensejar compreensão de sentidos individuais e coletivos particulares à localidade, juntamente à contextualização de sistemas interpretativos e nexos causais sobre a epidemia e a memória desse adoecimento em conjuntura sócio-histórica específica.

## A gripe espanhola na boca do sertão paulista

Localizada em um alto de serra no sudoeste paulista e criada freguesia em 1846 pelo afluxo de paulistas e mineiros em busca de terras para plantio e criação, Botucatu – bons ares em tupi-guarani (Ybytu-Katu) – tornou-se paróquia em 1849, vila em 1855 e cidade em 1876, mitificando-se em torno das alcunhas de "Terra dos Bons Ares", "Terra da Caridade" e de "Capital da Boca do Sertão" (Donato, 2008; Ribeiro, 2020; Ribeiro, Marques, Mota, 2020).

Como Boca do Sertão suas representações remontam a pouso, vereda, fronteira, estabelecendo-se nos Oitocentos como entreposto comercial de víveres e muares para o sertão do Paranapanema, e de mercado de negros escravizados trazidos de Minas Gerais, Bahia, Paraíba, Pernambuco e Rio Grande do Norte. A enaltecida localização geográfica em relação aos povoados limítrofes fez com que caminhos conduzissem ou atravessassem suas terras, tornando-se entroncamento de produtos entre a capital e o sertão paulistas (Donato, 2008; Ribeiro 2020; Ribeiro, Marques, 2021).

Nos primórdios da República, sob as benesses de expoentes do Partido Republicano Paulista (PRP), Botucatu recebeu estações da Estrada de Ferro Sorocabana, luz elétrica, sede de bispado, 5º Distrito Eleitoral, Delegacia Regional de Polícia, escola normal e grupo escolar, chegando a ter duas redes telefônicas – a Bragantina e a Sul Paulista (Magalhães, 1920; Antonini, 1985; Ribeiro, 2020).

Tais equipamentos, somados a um Hospital de Misericórdia – com administração e corpo clínico ligado à Ordem Maçônica – e diversas associações de benemerência conferiram destaque e singularidades a Botucatu.

Em período marcado pelas disputas entre a teoria bacteriológica e a teoria miasmática (por vezes justapostas) e pelo higienismo, em que topografia, clima e circulação dos ventos garantiriam salubridade urbana (Costa, 2014; Mantovani, Marques, 2020), Botucatu se autoproclamou livre de doenças pelos seus "bons ares" e pelo aparato médico-sanitário-filantrópico disponível (Ribeiro, 2020; Ribeiro, Marques, 2021).

Se Botucatu despontara como capital de extensa região do estado de São Paulo e norte do Paraná e como cidade mais populosa do planalto ocidental, com cerca de trinta mil habitantes ao final da década de 1910, a explosão demográfica e os conflitos inerentes à urbanização e à acumulação de capital revelaram, todavia, questões sociais e sanitárias irreconciliáveis (Antonini, 1985; Iyda, 1979).

Nesse estado de coisas, Botucatu alcançou 1918 tecida nas disputas e arranjos de suas forças sociais (o PRP, a Igreja católica e a Maçonaria) e sob os auspícios da paulistanidade, com a interiorização de aparato médico-sanitário e a instalação de mais um órgão público em suas terras: a Delegacia Estadual de Saúde (Ribeiro, 2020).

Braço do Serviço Sanitário paulista no interior do estado, a Delegacia Estadual de Saúde de Botucatu agregou interesses de autoridades públicas, elites locais e capitalistas na ocupação do Extremo Sertão paulista. De larga abrangência territorial, a delegacia serviu como solução coletiva de defesa sanitária nas fronteiras entre a "Capital da Boca do Sertão" e a palustre Zona Noroeste, então tomada pela leishmaniose e pela malária (Ribeiro, Marques, 2021; Ribeiro, 2020; Ribeiro, Marques, Mota, 2020).

Na primavera de 1918, enquanto a cidade se entretinha com seu aparato médico-sanitário-filantrópico e com a organização do Tiro de Guerra, do Escotismo e da Cruz Vermelha, chegou às suas terras a notícia de misteriosa enfermidade que corria o mundo (Ribeiro, 2020). Em fonte tipográfica minúscula e em meio aos relatos de geada, febre aftosa e lagarta-rosada que atacavam a cidade, uma nota esparsa em *O Correio de Botucatu* chamou a atenção:

> Meia centena de bravos, partidos na direção da refrega, cai em caminho, na devastação de uma moléstia ignorada a princípio e que se descobre ter irrompido devido às condições anormais dos países europeus. É a guerra, a guerra que traz, no seu clássico cortejo, a fome e a peste ... Agora é a horrível epidemia da 'influenza espanhola' que se abate sobre os navios da nossa esquadra em caminho e ceifa de início mais de cinquenta vidas preciosas e robustas (A Mosca, 28 set. 1918, p.2; destaque no original).

Nas semanas subsequentes, a quietude de *O Correio de Botucatu* sobre o galope epidêmico na capital paulista e na região tencionando um suposto controle sanitário, traía-se pelas publicações de serviços médicos e remédios, condolências, textos funestos e relatos da doença em várias localidades, em pequenas notas no miolo do jornal (Ribeiro, Marques, Mota, 2020).

Ao leitor atento, contudo, uma notícia não escaparia: o destaque conferido aos moradores do afastado e superpovoado bairro da Estação, pelo redator do jornal Levy de Almeida que, se colocando como porta-voz dessa população, fez a seguinte denúncia:

> ‘O perigo que está iminente ninguém calcula, pois no caso de se manifestar qualquer moléstia infecciosa, a sua propagação [no Bairro da Estação] seria inevitável, urgindo que a Câmara de acordo com os funcionários da higiene, dê ao caso a solução mais pronta e eficaz. O Sr. Dr. Waldomiro, atendendo à representação, verificou a procedência da queixa e, segundo sabemos está agindo seriamente, pois o caso requer a máxima atenção dos que têm, sobre os seus ombros, o pesado encargo de zelar pela saúde pública.’ A Câmara naturalmente, secundará os esforços da nossa autoridade sanitária, promovendo os meios necessários para a solução desse perigoso caso. Os habitantes do populoso Bairro [da Estação] é que não podem continuar sob a iminência desse perigo, importunados ainda, por uma exalação pestilenta e perseguidos por nuvens de pernilongos (Almeida, 19 out. 1918, p.2; destaque nosso).

Levy deixava claro: o que a segregação urbana separara e abandonara, a epidemia reuniria, ainda que as primeiras vítimas fossem os moradores do bairro da Estação.

Em 21 de outubro de 1918, apenas dois dias após o alerta de Levy de Almeida, a epidemia tomou seu lugar oficialmente em Botucatu. E como era esperado grassou impiedosamente entre os depauperados da cidade. Na edição de 26 de outubro de 1918, enquanto a “Grippina” foi capa do jornal *O Correio de Botucatu*, a seção “A Mosca” denunciou a incúria da municipalidade diante da intocável rotina citadina que seguia com colégios particulares, casas de moda, alfaiatarias, armazéns e circo abertos, oferecendo serviços e agradecendo a preferência:

> Depois da casa arrombada, trancas à porta. Foi sempre, esse, o critério adotado pela nossa imprevidência, diante de uma calamidade possível. Até agora, segundo me parece, afora a polvorosa em que andam os funcionários da higiene, nada se fez no sentido de isolar a população no caso de se manifestar, epidemicamente, a traiçoeira moléstia que é hoje o espantalho de outras terras. E por quê? Ora porque!... Porque não se quis fazer: porque manda quem pode e está acabado ... (A Mosca, 26 out. 1918, p.2).

Para “A Mosca”, a inação da municipalidade denunciava interesses comerciais e propósitos políticos que, ao se sobreporem ao bem da população, deixaram a cidade desnuda diante da epidemia. Porém, em meio aos zunidos das delações, uma inexplicável suspensão de circulação abateu-se por dias sobre *O Correio de Botucatu*.

Em 9 de novembro de 1918, após vários dias em silêncio, *O Correio de Botucatu* rompeu o período afônico com estrondosa capa: Levy de Almeida, o redator do jornal que meses antes conclamava os filhos da terra à cruzada sanitária, fora levado pela gripe espanhola, aos 33 anos de idade (Editorial, 9 nov. 1918; Ribeiro, Marques, Mota, 2020).

A morte de Levy, assinalada em letras garrafais na capa de *O Correio de Botucatu* e “considerada um acidente, um sinal de impotência ou de imperícia que é preciso esquecer”

(Ariès, 1990, p.791), atualizou-se no interior do luto familiar e do fenômeno coletivo epidêmico, tornando-se inflexão na linha editorial do periódico.

Na edição de 16 de novembro de 1918, em editorial demonstrando "acerto de contas" com a cidade, a família de Levy de Almeida, proprietária de *O Correio de Botucatu*, publicou reclamações sobre a inoperância sanitária, enalteceu as senhorinhas da Cruz Vermelha e os escoteiros e nomeou os que não abandonaram as alcovas dos gripados e, com mágoa, denunciou sobrenomes que, com medo do contágio, se negaram ao socorro (Editorial, 16 nov. 1918).

Na elaboração das sensações e das representações da memória e do luto e do extenso campo de significados que os envolvem, a família Almeida tomou para si o espírito crítico de "A Mosca" que seguira para o túmulo com Levy, em seu último voo, revelando ao leitor assíduo do jornal que o inseto servia de pseudônimo ao redator falecido. Sem a máscara do pseudônimo utilizado por Levy, *O Correio de Botucatu* abraçara publicamente os novos rumos do jornal.

Entendendo que o sofrimento pode repugnar e seduzir, despertar gestos de assistência ou sentimentos de compaixão (Farge, 2011, p.19), enquanto o luto transpassava os dias, a morte de Levy foi adquirindo caráter inefável nas páginas do periódico: ele já não morrera de gripe, mas de complicações cardíacas.

A glorificação ao seu espírito caritativo dava-lhe contornos de mártir, e a exaltação à sua memória tornou-se mote para a arrecadação de fundos para o Asilo dos Morféticos da cidade, que passava invariavelmente por apuros financeiros. Redimia-se a memória do boêmio (Ribeiro, 2020):

> Assistência aos Morféticos (Nossa subscrição) Levy de Almeida não se enganou quando apelou para a generosidade do público em favor da 'Assistência aos Morféticos'. Todos os que compreendem a nobreza desse gesto de filantropia têm vindo em nosso apoio. O sr. José Baptista de Faria angariou na fazenda S. João, onde é administrador, a quantia de 115$000, sendo 50$000 para a Misericórdia. Está aí um gesto nobilitante e que todos os senhores fazendeiros poderiam imitar (Assistência..., 30 nov. 1918, p.2; destaque no original).

Com o declínio epidêmico, em edição de 23 de novembro de 1918, *O Correio de Botucatu* assumira publicamente o que se inclinara a fazer desde a morte de Levy de Almeida: perpetuar a memória daqueles que seriam os beneméritos da cidade, pois "que criaram e mantiveram o hospital conquistaram lugar de destaque pela sua ação enérgica e dedicada" (Editorial, 23 nov. 1918, p.2).

Resplandecendo uns e ofuscando outros, silenciando particularmente a participação da Loja Maçônica Guia do Futuro e de seus membros em detrimento de elogios às autoridades eclesiásticas e às instituições católicas, as forças sociais da cidade rearranjavam-se após os padecimentos da população pela gripe espanhola. E a fé, em suas distintas representações e expressões, surgia como gesto político renovado em sua conjuntura (Certeau, 2014).

Com páginas dedicadas a listar os mortos pela epidemia informados pelo Cartório de Registro Civil da cidade, *O Correio de Botucatu* publicou em 14 de dezembro de 1918 contagem quase igual aos 55 óbitos divulgados pelo Serviço Sanitário paulista, que apontara a notificação de 1.016 casos de gripe em Botucatu entre seus aproximadamente trinta mil habitantes (Editorial, 14 dez. 1918; Ribeiro, 2020; Ribeiro, Marques, Mota, 2020).

Entre 1.016 adoecidos, 371 foram socorridos no Hospital de Gripados – instalado às pressas no prédio da Escola Normal Cardoso de Almeida, às expensas da Prefeitura e destinado aos doentes reconhecidamente pobres –, e outros 281 foram socorridos em domicílio com medicamentos e víveres, chegando-se assim ao socorro de 64% de moradores depauperados (Meyer, Teixeira, 1920, p.321; Alves, 1918, p.196; Ribeiro, 2020, p.147).

O que nos leva a concluir que a maioria das vítimas em Botucatu se encontrava em más condições de vida e em regiões desprovidas de salubridade, quadro agudizado pela borrasca epidêmica. Tal cenário de incúria também foi apontado por Bertolli Filho (2003) em seus estudos sobre a gripe espanhola na capital paulista.

Na construção de narrativa sobre a Comissão de Socorros, contudo, o Hospital de Gripados surgiu enaltecido pelos dirigentes públicos e pela imprensa como grande obra de caridade, ainda que vestígios históricos tenham apontado seu claro caráter asilar, destinando-se a internar pontualmente doentes pobres para dirimir focos de contágio na cidade.

A instalação do Hospital de Gripados também evidenciou novo arranjo na tecnologia assistencial botucatuense: ao poder público caberiam ações mais gerais, e à filantropia, ações pontuais, diferenciando-se da atuação caritativa das obras de benemerência locais e acenando contexto propício para uma transição que já se fazia sentir entre as associações botucatuenses (Ribeiro, 2020).

Anotações de párocos em Livro de Óbitos da Cúria de 5 de setembro de 1915 a 1º de agosto de 1926 sobre falecimentos ocorridos em Botucatu entre 1918 e os primeiros anos da década de 1920, tendo como *causa mortis* a gripe espanhola e febres diversas, nos revelam que epidemias se sobrepuseram e se entrelaçaram à carestia no período estudado. Também nos apontam as dificuldades do consolo aos fiéis, considerando a vastidão territorial do bispado de Botucatu.

Dificuldades também sentidas pela Delegacia de Saúde de Botucatu diante do enorme território sob sua responsabilidade: a dispersão populacional, as impossibilidades de deslocamento e o colapso do Serviço Sanitário no transcorrer da gripe espanhola fizeram com que somente depois de declinada a epidemia na capital fossem enviados e recebidos remédios e funcionários para as franjas do estado (Ribeiro, 2020).

Relevante ressaltar os constantes embates entre forças sociais, redes clientelares e redutos políticos que configuraram impasses entre as instâncias oficiais envolvidas com a saúde pública (municipalidade e Delegacia de Saúde) em Botucatu.

O descompasso nas ações impetradas antes e durante o evento epidêmico na cidade deslindam contradições e resistências no projeto de sanear o estado bandeirante, permitindo entrever limites estruturais, fragilidades e equívocos na implementação do projeto sanitário paulista – em especial o de sua interiorização (Ribeiro, 2020; Ribeiro, Marques, 2021; Bertucci, 2004).

Em sessão da Câmara Municipal de 15 de janeiro de 1919, foi lido o relatório de 1918 pelo prefeito de Botucatu. Entremeado por elogios às condutas de personalidades e de instituições da cidade na Comissão de Socorros aos gripados, criteriosamente escolhidas para figurar na memória oficial e apagando doenças e riscos sanitários em bairros periféricos, assim surgiu a cidade no novo ano:

> Não fosse a terrível pandemia de gripe espanhola, que com caráter verdadeiramente virulento invadiu em novembro último a nossa cidade, poderíamos afirmar que o estado sanitário de Botucatu continuava a ser dos melhores, pois até aquela época era bastante lisonjeira a saúde pública de todo o município (Kuntz, 1919, p.29).

Na virada para 1919, foi organizado por jovens da elite o baile do "enterro do ano velho de 1918", no badalado Club 24 de Maio. Talvez, poucos vestígios espelhem mais a "multiplicação dos efeitos pela rarefação dos meios" (Certeau, 2014, p.146).

Metamorfoseado e capilarizado em falas e fatos corriqueiros e até mesmo banalizado, o evento epidêmico esvaziou-se do sentido trágico, permitindo a retomada da cidade na retroalimentação de seus mitos de origem como "Terra dos Bons Ares", "Capital da Boca do Sertão" e "Terra da Caridade" (Ribeiro, 2020).

Nas engrenagens do poder da memória e da memória do poder, a gripe espanhola em Botucatu desintegrou-se para integrar-se ao tempo tal qual a poeira de seus mortos.

## Nas fímbrias da memória

Nos anos posteriores à epidemia de gripe espanhola em Botucatu, verificou-se nos vestígios históricos coligidos a demonstração de uma nova prática: o ato de esmolar e dar esmolas no cemitério da cidade no Dia de Finados. Para tanto, faz-se importante reter o aumento de visitas ao cemitério desde 1919 decorrente das mortes epidêmicas em 1918, como também as camadas sociais às quais pertenciam os gripados ali enterrados e que renderam as esmolas de suas famílias:

> Ainda não se foi apurado o quantum das esmolas angariadas por diversas senhorinhas, por ocasião das visitas ao Cemitério Municipal no dia de Finados, em benefício do Asilo de Mendicidade e da Misericórdia Botucatuense. Sabe-se, entanto, que já foi apurada quantia que monta, mais ou menos, em 900$000. É este um belo auxílio que o nosso povo oferece àquelas casas de caridade (Para casas..., 6 nov. 1920, p.2).

Em cotejamento de fontes, foi possível entender que a prática de esmolar no cemitério em Dia de Finados foi não só promovida nas edições de *O Correio de Botucatu*, mas também se fez presente em ata de 28 de outubro de 1921 da irmandade católica feminina Comunhão Reparadora.

Padre Euclides, pai espiritual das senhoras e senhorinhas da Irmandade Comunhão Reparadora, encontrava-se às voltas com a inauguração do afastadíssimo Asilo de Mendicidade. Para a realização da obra de caridade sob sua proteção e destinada a recolher pobres, enfermos e indigentes que desfilavam sobremaneira pelas ruas de Botucatu após a carestia que acompanhou a epidemia de gripe espanhola, esmolar se fazia fundamental:

> tomou a palavra nosso bondoso diretor [padre Euclides] que convidou a todas as associadas para angariar donativos à porta do cemitério nos dias 1º e 2 de novembro em benefício da Santa Casa de Misericórdia. Todas mostraram-se prontas para trabalharem para este fim tão nobre e justo (Morato, 1928, p.11).

Em sofisticada produção de sentidos, a esmola passara a cumprir papel de reificação da memória do morto e suporte de sua lembrança, reunindo em seu ato as almas de vivos e falecidos pela salvação da caridade, inclusive os que, ainda no plano terreno, sôfregos pelo delírio febril da gripe espanhola, não teriam cumprido o sacramento católico da confissão de seus pecados e recebido o esperado perdão:

> A verdadeira ligação está em nossas orações e nas boas obras que nos seja possível praticar em benefício de suas almas. A esmola constitui um dos trabalhos meritórios que poderemos fazer em seu nome [dos mortos] e suas almas manifestarão o prazer, levando ao trono do Altíssimo a ação boa por nós praticada em sua memória, especialmente quando esta esmola tem por fim aliviar o sofrimento da pobreza. O Asilo de Mendicidade, em vésperas de funcionamento, necessita do concurso dos bons corações que felizmente contam-se em grande número nesta cidade. No dia de Finados diversas pessoas encarregar-se-ão de angariar donativos em benefício dos pobres do Asilo para a compra do necessário à instalação definitiva dessa piedosa instituição. O óbolo que cada um depositar na bolsa, será como a representação dos esforços dos próprios mortos para se manter a utilíssima casa de caridade e os entes queridos de nossa estima conduzirão esse ato de real aproveitamento, até Deus, intercedendo por nós e fazendo derramarem-se bênçãos sobre nossas pessoas (A Cidade..., 1º nov. 1922, p.1).

Para Philippe Ariès (1990, p.329-330), o pensamento da morte se distribui pelo tempo da vida e estabelece uma relação estreita entre o bem-viver e o bem-morrer na sensibilidade coletiva e nos limiares de tolerância da dor. Nesse sentido, a memória dos mortos torna-se capaz de se sobrepor ao não dito a partir de representações coletivas e ressignificações pessoais (Le Goff, 2003; Benjamin, 1987).

A gripe espanhola, assim revivida e articulada em lembranças pungentes carregadas por indivíduos e instituições, seguiu atravessando os anos em Botucatu rememorada em constantes alertas sobre a pestilência e a necessidade de um estado de prontidão não só da população, mas do poder público e das associações de benemerência, revelando o medo de uma nova onda epidêmica na cidade.

Da redação de *O Correio de Botucatu* partia não só a atenção sobre o cemitério da cidade, anúncios de funerárias e taxas de serviço, mas também a preocupação com o lixo e com as moléstias incessantes:

> Botucatu, que se poderia considerar verdadeiro sanatório tal a excelência de seu clima, é um lugar dos mais perigosos para habitação, para residência. Nem em Salto Grande, Anhembi, Porto Feliz, Tietê e Porto Martins, com as suas maleitas periódicas, os seus habitantes correm tanto perigo! Nós os habitantes de Botucatu, não temos amor à vida... Parece até incrível! E se não, nos expliquem como em um clima nestas condições, um dos melhores de S. Paulo, inúmeros casos de febres de mau caráter surgem aqui todos os anos? (Sejamos previdentes, 24 mar. 1920, p.2).

O enterramento de corpos despossuídos de bens e a melhoria no deslocamento de cadáveres para a última morada revelam como a ideia e a memória da morte e da gripe espanhola encontravam-se vívidas, circulares e porosas no cotidiano da cidade pós-epidemia:

> Além de outras necessidades temos uma a resolver, com uma certa urgência – dotarmos a cidade com um carro fúnebre. Cidade grande, de extensas ruas, montanhosa, e de cemitério localizado num de seus extremos – o transporte dos mortos exige, às vezes, grandes sacrifícios dos que os levam para o eterno repouso – quando facilmente se poderia tornar a todos nós materialmente menos pesada a tarefa, a aquisição de um coche fúnebre. Em dias chuvosos, é o caridoso ato dos que acompanham os féretros, penosíssimo. É verdade que a Casa de Misericórdia, às vezes, para certos enterros, cede uma sua carrocinha fúnebre… mas esta tão modesta, tão imprópria para figurar em certos enterros. Necessitamos de coisa muito mais decente. Aqui temos tanta gente caridosa e de recursos, que não se negariam em levar avante a nossa ideia… (Além, 24 mar. 1920, p.3).

Meses depois do apelo, a resposta veio pela criação da Sociedade de São Tobias, destinada ao enterro da população miserável falecida em leitos da Misericórdia Botucatuense:

> Piedosos cavalheiros desta cidade, há poucos dias, reunidos, fundaram a Sociedade de S. Tobias, cujo fim humanitário é o de fornecer caixões para o enterramento dos pobres que falecem na nossa Casa de Misericórdia. Como é sabido, o cadáver desses indigentes era levado à cova, até então, sem caixão etc. Este fato, que não está de acordo com os sentimentos piedosos daqueles cavalheiros, vai desaparecer, pois cada um destes, se compromete a fornecer ao pobre falecido, caixão e o mais que for necessário. Para isso, não haverá sorteio entre os sócios de tão humanitária associação, pois cada um receberá um número de ordem, e, a proporção que se forem dando os falecimentos, os sócios, em ordem, irão pagando as despesas de enterramento do indigente (Sociedade de S. Tobias, 20 out. 1920, p.2).

A atenção e tensão sobre a morte e o morrer também foram externadas pela municipalidade em atas da Câmara Municipal de Botucatu. Por quase um ano, nas sessões de 20 de maio de 1919, 20 de dezembro de 1919 e 12 de março de 1921, discutiram-se propostas sobre alterações de normas dos túmulos, mudanças no regimento do cemitério e autorização para funcionamento de empresa funerária na cidade (Botucatu, 1919a, 1919b, 1921).

Na esfera da produção de sentidos e apreensões sobre a lembrança da dor, o diário de João Valério de Oliveira traz em seu comovente testemunho e energia da memória declarativa (Ricoeur, 2007, p.504).

Homem pardo e nascido em Porto Feliz, residente e escrivão de paz e oficial do Registro Civil no distrito de Bom Jesus do Ribeirão Grande,[1] João Valério encontrava-se encarcerado no referido distrito na época da epidemia de gripe espanhola (Godoy, 2001, p.28) e terminou seus dias no Asilo de Mendicidade Padre Euclides em Botucatu.[2]

A antecipação do epitáfio, na folha de rosto de seu diário, evoca em tom quase fantasmagórico o cumprimento de promessas adiadas não mais àquele que escreveu a história, mas aos que farão a história (Ricoeur, 2007, p.506) pela perpetuação de sua existência por meio da preservação de suas lembranças na força da palavra:

> Servirá este livro para lembrança da família ou recordações com modinhas, lundus e recitativos para violão etc. e também suas folhas numeradas e rubricadas, por mim escrito datado e assinado. Ribeirão Grande, 7 de agosto de 1896 (Oliveira, 1941, contracapa).
> …

> Em memória de mim, porque espero que a minha vida neste mundo de tantas ilusões, vaidades, enganações, traições e falsidades, está perto de eu deixar… Que zelem deste livro e respeitem; porque está destinado à leitura a todos, pra ficarem cientes mais ou menos o que eu passei durante e desde o meu casamento, com quem até hoje. Por isso, proíbo-te a violência ou estragos… Ouviram?! O meu espírito velará, aqueles que fizer o contrário… Por ser verdade do que tudo está escrito. Dato e assino. Botucatu, 30 de janeiro de 1925 (Oliveira, 1941, contracapa).

As páginas escritas por João Valério sobre suas perdas familiares para a gripe espanhola em Bom Jesus do Ribeirão Grande – zona rural de Botucatu – e as aflições para sobreviver à carestia e ao desemprego ao mudar-se para a zona urbana do município diante das greves e sedições de 1924 e 1932, deixam rastros para hipótese de que o distrito de Bom Jesus do Ribeirão Grande tenha sido dizimado pela gripe espanhola em 1918 e 1919.

A dor apreendida e revisitada nas memórias de João Valério nos enseja a discussão sobre raça, pobreza e desigualdades regionais em contexto epidêmico. Seu testemunho nos lança ao debate das permanências sócio-históricas como lugar estruturante das múltiplas dimensões do viver, adoecer e morrer tanto nas práticas e discursos médico-sanitários, quanto no âmbito da assistência, abarcando singularidades do sofrimento e do constituir-se doente no conjunto da sociedade e expressando-se como testemunho de um tempo coletivo (Schraiber, 1993; 1997; Marques et al., 2018):

> Na ocasião da Gripe, Adalgisa filha de Francisco faleceu dia 6 de novembro de 1918, e no dia 12 do mesmo mês faleceu Luiz o irmão com 17 anos mais ou menos a irmã uns 18 anos, daí a 3 dias faleceu a prima Rita filha do finado Antonio Velbido, 25 de março de 1919 faleceu mais uma menina filha de Francisco … (Oliveira, 1941, fl.27).

Envolto em lendas e mistérios, o desaparecimento do povoado de Bom Jesus de Ribeirão Grande possui várias versões entre cronistas locais: praga rogada por padre, disputas políticas entre expoentes do PRP na região, abertura de novas estradas desviando a rota de viajantes e a "febre" espanhola – esta última interpretada como castigo divino devido à maldição de um pároco (Godoy, 2001).

Um relatório anual apresentado pelo prefeito Nicolau Kuntz à Câmara Municipal em 15 de janeiro de 1920, chama atenção, todavia, sobre o aniquilamento de vidas pela gripe espanhola no transcorrer de 1919, na zona rural de Botucatu:

> O distrito do Espírito Santo do Rio Pardo [nele incluso Bom Jesus do Ribeirão Grande] que em 1918 não havia sido atingido pela terrível epidemia, em 1919 foi também alcançado pela gripe espanhola, com caráter violento, fazendo inúmeras vítimas (Kuntz, 1920, p.89).

Narrativas que, quando aferidas com as memórias de João Valério e com o relatório da municipalidade sobre os inúmeros óbitos ocorridos nesse Distrito, remetem à invisibilidade de vidas ceifadas pela gripe espanhola.

Considerando que a memória se tece nas relações entre sujeitos históricos e o seu cotidiano, dialeticamente mobilizando ações e sendo mobilizada por elas (Le Goff, 1984), a análise em curso tem nos revelado outra peculiar rememoração: a lembrança e a evocação da estratégia assistencial no socorro aos gripados durante as conflagrações de 1924, 1930 e 1932, em Botucatu e na capital paulista.

No caso do movimento revolucionário de 1924, deflagrado na cidade de São Paulo em 5 de julho de 1924 como segunda revolta tenentista, capital e demais localidades paulistas sofreram por 28 dias ataques incessantes de canhões, tanques de guerra e bombardeios pelas tropas legalistas. Tal conflagração resultou em inúmeros deslocamentos em massa, aproximadamente 4.846 feridos e entre 503 e mil mortos (Assunção, 2015).

O cenário de guerra esmiuçado no editorial de *O Estado de São Paulo* de 18 de julho de 1924 não só carrega detalhes da rotina terrificante daqueles dias, mas a rememoração e reconvocação das estratégias assistenciais utilizadas no socorro aos gripados da espanhola.

Nessa edição, a Cruz Vermelha paulistana, cujo papel assistencial destacou-se no socorro aos gripados de 1918, surge recolhendo cadáveres para o Araçá, levando doentes ora para abrigo na Escola Normal Caetano de Campos ora para a Santa Casa.

A Igreja católica, na figura do arcebispo metropolitano dom Duarte, aparece na organização de préstimos à população refugiada por meio dos jovens da União dos Moços Católicos, da Congregação dos Filhos de Maria e da Associação do Apostolado Coração, sendo acompanhada pelo pastor do Templo Metodista que noticia seu salvo-conduto para levar víveres ao seu rebanho.

Os escoteiros, tal qual auxílio prestado outrora às municipalidades para os acamados da gripe espanhola (Ribeiro, 2020), fulguram no episódio de 1924 como mensageiros de cartas, receitas para aviar, entrega de remédios e junto à associação dos empregados do comércio.

Ainda nessa mesma edição, há relato sobre um hospital de isolamento providenciado rapidamente para caso de epidemias, considerando-se a quantidade de cadáveres aguardando enterramento e o intenso deslocamento das massas em trens para o interior, vagando pelas ruas ou mesmo pedindo abrigo em escolas e grupos escolares:

> Conforme noticiamos, desde o dia 6 do corrente até o dia 16, foram sepultadas no cemitério do Araçá 122 vítimas da revolução, verificando-se pela lista que reproduzimos, serem elas, na sua absoluta maioria, pessoas da população civil. O serviço no cemitério do Araçá está sendo feito com toda regularidade, apesar das dificuldades que só em aparecer numa 'ocasião anormalíssima como a que atravessamos. ... Os sepultamentos estão sendo feitos na nova área do cemitério, adquirida ainda por ocasião da gripe em 1919'. Todas as sepulturas são numeradas, não se tendo feito o uso de vala comum (No Araçá, 18 jul. 1924, p.2; destaque nosso).

Por sua vez, em Botucatu – rota de passagem das forças revolucionárias pelo interior paulista, sob o comando de Cabanas (2014) –, o intenso envolvimento de parte da população no movimento de 1924 torna-se patente em comunicado à população publicado no jornal local.

O comunicado "Ao Povo", estampando a capa de *O Correio de Botucatu* de 26 de julho de 1924, revela a instalação de um governo revolucionário na cidade sob a liderança de Gastão Pupo, enquanto as páginas subsequentes descerram o movimento de tropas "sediciosas" pelas estações da ferrovia Sorocabana e o temor local em sofrer bombardeio de forças governistas.

Enquanto isso, a Cruz Vermelha de Botucatu, criada inicialmente sob os agouros da entrada do Brasil na Primeira Guerra Mundial em 1917, com atuação marcante nas enfermarias da gripe espanhola e dissolvida após o encerramento do Hospital de Gripados, em dezembro de 1918 (Ribeiro, 2020), é rememorada, reconvocada e reativada na conflagração de 1924. Assim, a Cruz Vermelha ressurge como estratégia assistencial de emergência pela Comissão de Socorros formada na cidade, diante dos embates entre revolucionários e tropas governistas.

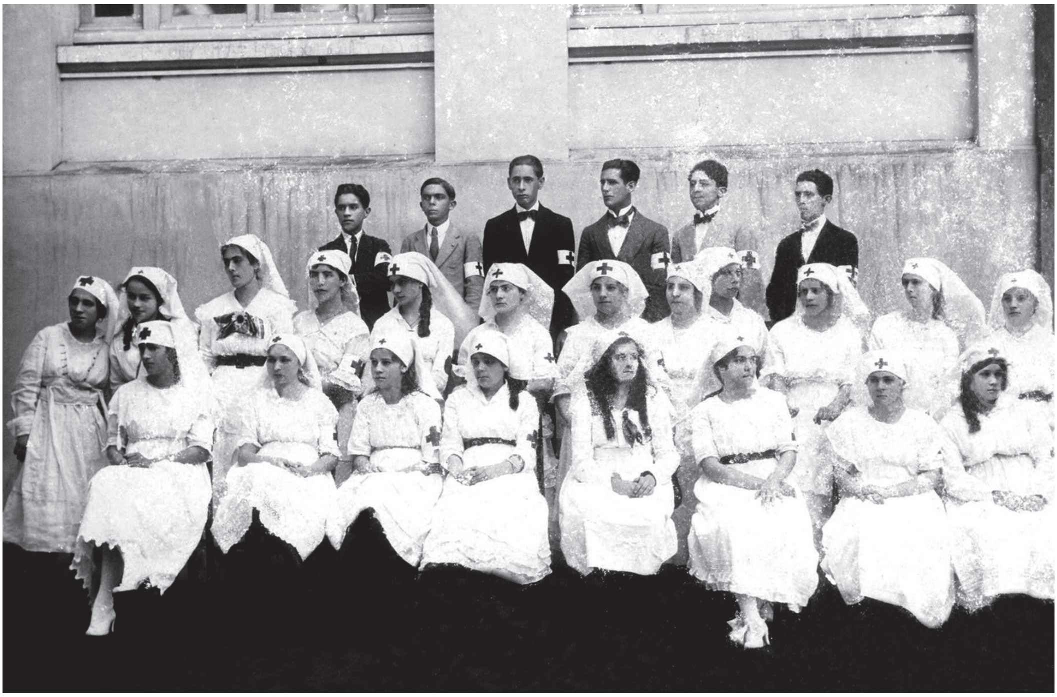

Figura 1: Cruz Vermelha de Botucatu em 1924 (Rocha, 1924)

Anos depois, em meio às turbulências políticas e sociais no país – especialmente em São Paulo –, não por acaso, a edição de 30 de outubro de 1930, *O Estado de São Paulo* presta homenagens à Cruz Vermelha Brasileira. O jornal rememora seu eficiente socorro na gripe espanhola, na conflagração de 1924 e nos combates durante a destituição de Washington Luís da Presidência da República e na instauração do governo provisório que alçou Getúlio Vargas a presidente do país.

Segue nota sobre o comboio sanitário organizado pela Cruz Vermelha e enviado a Botucatu para a instalação de um hospital de evacuação no Hospital de Misericórdia Botucatuense:

> Durante os dias de apreensão que S.Paulo viveu, muitas foram as instituições que prestaram assinalados serviços nessa emergência. Entre estas é de justiça destacar a Cruz Vermelha Brasileira, em São Paulo.
> Tal como já fizeram em ocasiões anteriores, por ocasião da gripe e da revolta de 1924 – a Cruz Vermelha, com a urgência que as circunstâncias reclamavam, organizou um serviço de socorros que não teve uma falha. Assim é que aquela instituição conseguiu, em breve espaço de tempo, ter à sua disposição um comboio sanitário modelar, para o transporte de feridos, das linhas de frente para os hospitais: enviou para Botucatu um corpo de 9 médicos, 10 enfermeiros e 20 estudantes de medicina, pessoal esse que

> seguiu para aquela localidade acompanhado da presidência da Cruz Vermelha, Sra. D. Antonia F. de Souza Queirós, a qual providenciou pessoalmente para a instalação de um hospital de evacuação.
> Esse hospital foi instalado na Santa Casa de Botucatu [Hospital de Misericórdia] e dispunha de um completo aparelhamento. Como terminasse a situação de anormalidade por que passou o Brasil, não foi necessária a utilização do referido estabelecimento, tendo regressado ontem, de Botucatu, a sra. presidente e os médicos, enfermeiras e estudantes que formavam a turma.
> Além dessa organização, a Cruz Vermelha poderia hospitalizar e prestar assistência, nesta capital, a algumas centenas de pessoas caso se tornasse necessário: tanto em sua sede como no hospital que possui em Indianápolis foram preparadas camas e material em abundância (Cruz…, 30 out. 1930, p.3).

Décadas depois, a passagem da gripe espanhola em Botucatu é novamente reconvocada nas palavras de médico Olívio Stersa ([199-?]).

Com o intuito de atribuir distinção de heroísmo, caridade e perpetuação de existência ao Hospital de Misericórdia, ainda que essa instituição não tenha participado do socorro aos gripados (Ribeiro, 2020, p.143-144), a memória da epidemia pavimenta a exaltação da assistência local em anos mais recentes:[3]

> A Saúde Urbana de Botucatu
> ‘a Misericórdia Botucatuense, que, inaugurada a 8.12.1901, passou a servir a todo o Oeste Paulista, destacando-se em todos os eventos ocorridos, tais como a gripe espanhola, Revolução de 1932, até hoje prestando inestimáveis serviços’, abrigando nos seus primeiros anos de funcionamento a Faculdade de Ciências Biológicas de Botucatu … (Stersa, [199-?]; destaque nosso).

Ao prefaciar a crônica de Almeida (1994) sobre o Hospital de Misericórdia Botucatuense, Stersa novamente apela a uma memória assistencial dessa instituição no socorro aos enfermos da gripe espanhola, em prol da salvação do hospital, que nos anos de 1990 passava por mais uma grave crise financeira:

> Em 1918, quando a “gripe espanhola” ceifou muitas vidas e trouxe grande pânico à comunidade o hospital foi retaguarda que prestou total assistência aos seus doentes.
> …
> Não podemos, entretanto, nos conformar com a situação [crise financeira da Misericórdia] e deixar que tudo vá se arrastando rumo ao desconhecido, sem procurar encontrar uma solução que se possa nos conduzir a melhores condições de atendimento (Almeida, 1994).

A rememoração de uma assistência às vítimas da gripe espanhola pelo Hospital de Misericórdia Botucatuense que não ocorrera ilustra as estratégias na resistência do estabelecimento de um lugar da memória entre as camadas do tempo e de forças atuantes em épocas históricas. Memória esta que dialeticamente guarda em sua constituição e fruição a relação entre o “não mais” – em seu caráter acabado, abolido, ultrapassado –, e o “tendo sido” que se designa em seu caráter originário, dinâmico, fluido e, por isso, indestrutível (Le Goff, 1984; Certeau, 2014, p.96; Ricoeur, 2007, p.505).

Resta-nos assim enfrentar o jogo dialético da experiência temporal e da operação narrativa (Ricoeur, 2007, p.17), pinçando rastros nas fímbrias do tempo. E, nessa perspectiva, perscrutar sentidos individuais e coletivos, interpretar a produção dos silêncios, ocultamentos e esquecimentos, enunciar outras vozes em suas dimensões políticas e afetivas em sua experiência e o direito de existir no espaço-tempo pela lembrança.

## Considerações finais

O presente estudo buscou apresentar como a memória da gripe espanhola permaneceu em Botucatu ao longo dos anos. Para tanto, pinçou sentidos e representações individuais e coletivas no viver social, entrelaçando-os às significações históricas e existenciais, articulando-os às especificidades locais e às idiossincrasias paulistas na conjuntura política e de saúde pública do período estudado.

O estudo investigou como a memória da gripe espanhola e de seu sofrimento foi reconvocada e transmutada ao longo do processo histórico em Botucatu, expressando-se na reconfiguração de ritos fúnebres e de estratégias assistenciais, mas também propondo um deslocamento epistêmico nos campos da história e da saúde coletiva.

Tal deslocamento epistêmico, assentado no corpo mediado pela experiência temporal de indivíduos e coletividades e compreendido historicamente por suas definições e funções anátomo-clínicas (Mota, Machin, 2018, p.421), defende que a epidemia de gripe espanhola não encontrou seu fim, mas sim incorporou-se às fibras da sociedade.

Nessa perspectiva, entende-se que a epidemia de gripe espanhola se capilarizou entre dimensões sociais, culturais, políticas e econômicas, modificando estruturas de poder e conformando-se como um evento histórico de longa duração.

Nessa medida, o trabalho confrontou particularidades com grandes modelos explicativos, articulando, na conjunção dos contraditórios, vestígios do passado e peças da memória no tensionamento das relações do presente e do não presente.

Desta feita, considera-se que as forças históricas que moldaram o século XX continuam a operar (Hobsbawn, 1995, p.562) e que urge avançar na complexificação das relações de poder e de sociabilidade implicadas nas febres conjunturais.

AGRADECIMENTOS

O presente trabalho é apoiado pelo Conselho Nacional de Desenvolvimento Científico e Tecnológico (CNPq).

## NOTAS

* Este artigo é desdobramento das discussões presentes na dissertação de mestrado *Entre alcunhas, altares e alcovas: a gripe espanhola na boca do Sertão Paulista. Botucatu, 1918* articuladas com atual pesquisa de doutorado, *Dos fios nas fibras, dos rastros nas fímbrias: a Gripe Espanhola no sertão paulista. Botucatu-SP, 1918-1938*.

[1] Povoamento pioneiro no sertão paulista e atualmente desaparecido, Bom Jesus do Ribeirão Grande chegou a despontar na região sediando casa de cadeia, comércio, igreja, cemitério e cartório. Ao final do século XIX foi elevado a distrito de paz do município de Botucatu, sendo incorporado ao também distrito de Espírito Santo do Rio Pardo (atual município de Pardinho) em 1910, quando então contava com três mil moradores.

[2] Na contracapa do “Diário de João Valério de Oliveira”, documento histórico não publicado, há ficha colada com inscrição manuscrita onde consta João Valério como homem pardo e viúvo, natural de Porto Feliz, sendo seus pais Isaías A. de Oliveira e Laura M. de Jesus. Consta ainda que deu entrada no Asilo Padre Euclides em 10 de agosto de 1939, falecendo em 25 de abril de 1941, aos 71 anos.

[3] O texto encontra-se datilografado e assinado, mas sem data, avulso e com grampos enferrujados na pasta “Misericórdia”, em acervo do Centro Cultural de Botucatu. É possível saber que o documento é posterior a 1990, pois o autor refere-se à estrutura do Sistema Único de Saúde (SUS) na cidade.
